# Eosinophilic Esophagitis With Subsequent Eosinophilic Colitis: Keeping a High Index of Suspicion

**DOI:** 10.7759/cureus.22073

**Published:** 2022-02-09

**Authors:** Leen Z Hasan, Eric Vecchio, Qian Wu, Steven A Goldenberg, Houman Rezaizadeh

**Affiliations:** 1 Department of Medicine, University of Connecticut Health, Farmington, USA; 2 Department of Gastroenterology and Hepatology, University of Connecticut Health, Farmington, USA; 3 Department of Pathology, University of Connecticut Health, Farmington, USA

**Keywords:** eosinophilic enterocolitis, eosinophilic gastroenteritis, eosinophilic colitis, eosinophilic esophagitis, eosinophilic gastrointestinal diseases

## Abstract

The prevalence of eosinophilic esophagitis (EoE) has significantly increased, while, in comparison, eosinophilic gastroenteritis and colitis remain rare entities. The diagnosis and management of eosinophilic gastrointestinal (GI) disorders can be challenging given the non-specific manifestations and variable treatment response. Symptoms refractory to initial therapies (e.g., proton pump inhibitors, dietary modifications, topical steroids) should raise suspicion for distal involvement of the GI tract. In this case report, we describe a patient with EoE with a subsequent diagnosis of eosinophilic colitis and symptom response to systemic corticosteroids. In addition, we review recent updates regarding the management of eosinophilic gastrointestinal disorders.

## Introduction

Eosinophilic gastrointestinal diseases (EGID) are a group of inflammatory conditions that cause the accumulation of eosinophils in the gastrointestinal (GI) tract without a known secondary cause [1]. Eosinophilic gastroenteritis (EGE) and eosinophilic colitis (EC) remain less common than eosinophilic esophagitis (EoE), with an overall prevalence of EGE and EC estimated to be 5 and 2 per 100,000 persons, respectively, compared to 25.9 per 100,000 persons for EoE [1-3]. Although the pathophysiology is poorly understood, a combination of genetics, environmental factors, and immune-mediated hypersensitivity to ingested allergens appears to play a role [4]. Diagnosis requires a high index of suspicion as symptoms are non-specific depending on the anatomical location and layer of the GI tract affected [5]. Recommendations regarding the diagnosis and management of EGE and EC are based on limited data and consensus [2,6]. In this article, we present a case of EoE with a subsequent diagnosis of EC.

This article was previously presented as a meeting abstract at the 2021 American College of Gastroenterology Annual Scientific Meeting on October 25, 2021.

## Case presentation

A 33-year-old male with a history of asthma, allergic rhinitis, and gastroesophageal reflux disease (GERD) was diagnosed with EoE at age 28. He initially presented with dysphagia and reflux symptoms, improving minimally after proton-pump inhibitor (PPI) therapy. No other GI or systemic manifestations were reported at that time. Initial esophagogastroduodenoscopy (EGD) demonstrated a ringed esophagus with two dominant rings in the upper and middle esophagus. A tissue biopsy revealed >50 eosinophils per high-power field (hpf) consistent with EoE. He received a three-month course of topical fluticasone (220 µg twice daily) with partial relief, in combination with dexlansoprazole 60 mg and an intermittent four-food elimination diet. A higher dose of swallowed fluticasone 440 µg twice daily was also trialed without response. He experienced persistent dysphagia, reflux, and choking sensation at times for which he required repeated EGDs and dilatations two to three times per year. Later in the course of his disease, he experienced chronic diarrhea and abdominal cramping raising the suspicion for EGE/EC.

**Figure 1:**
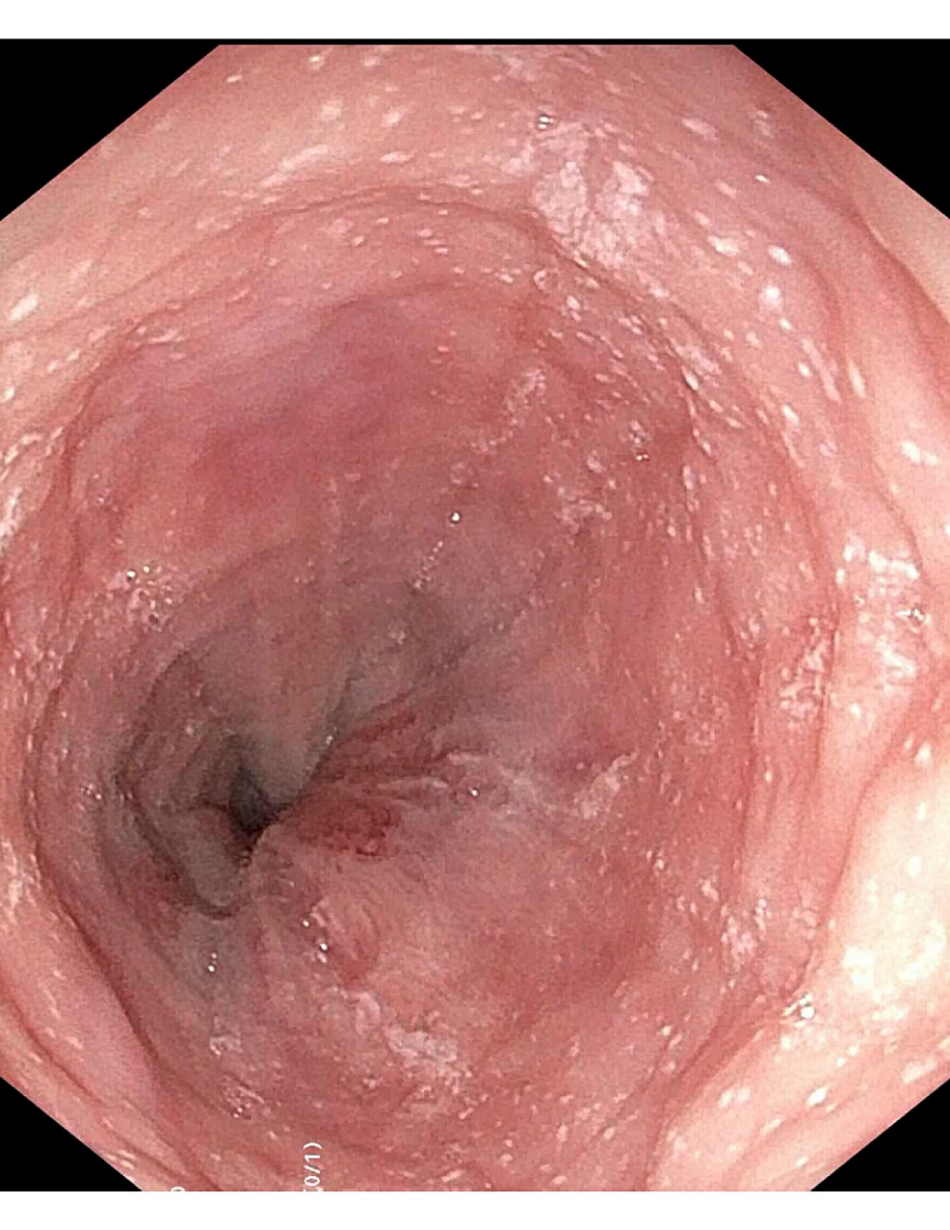
Gross appearance of the esophagus in a patient with eosinophilic esophagitis. Upper endoscopy showing distal esophagus with significant eosinophilic exudates.

**Figure 2:**
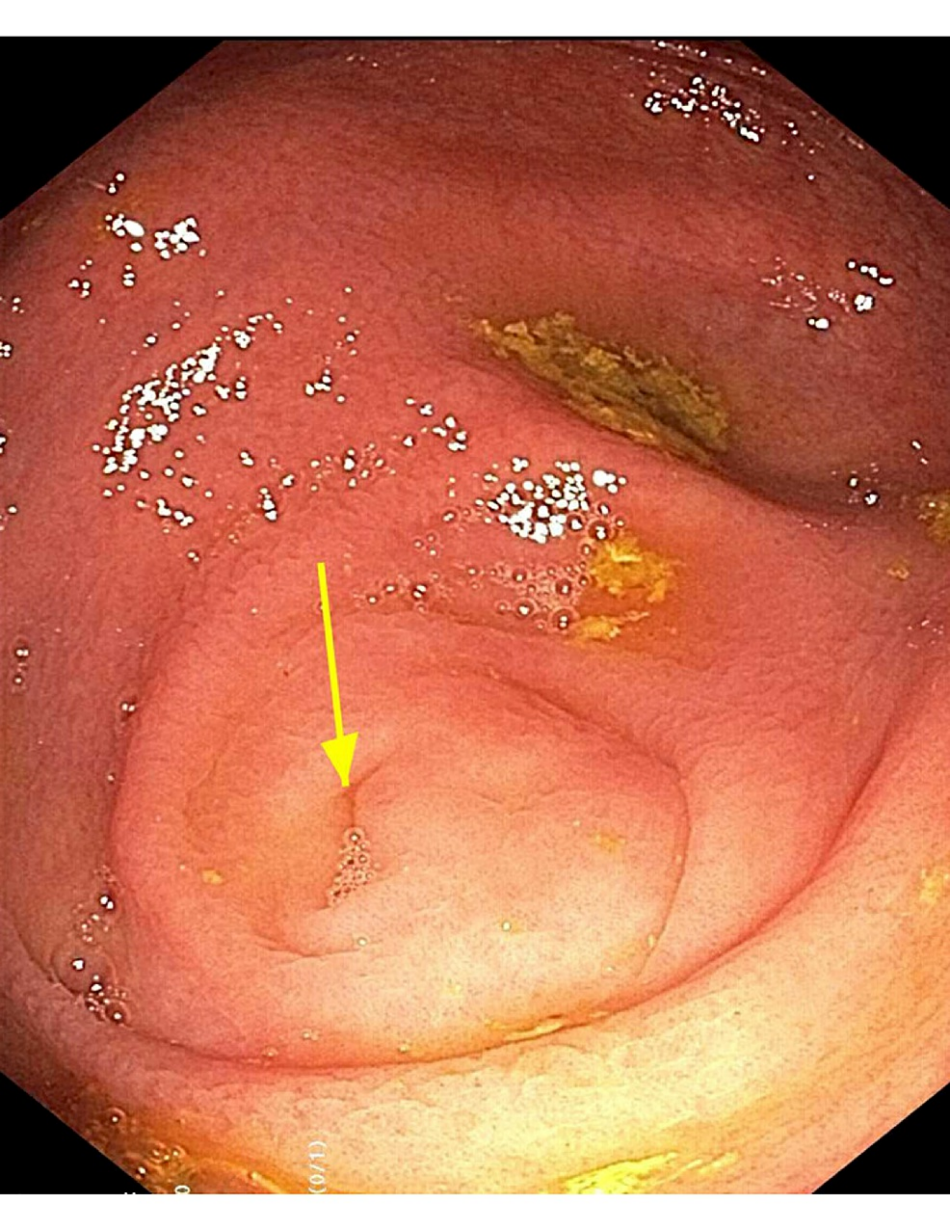
Gross appearance of the colon in a patient with eosinophilic colitis. Colonoscopy showing appendiceal orifice (yellow arrow) with mildly abnormal surrounding tissue notable for edema, vascular pattern changes, and granular mucosa.

A repeat EGD with a colonoscopy was performed. The EGD yielded a ringed esophagus with longitudinal furrows and exudates without strictures (Figure 1). Colonoscopy showed a 3-mm polyp in the sigmoid as well as granular mucosa and edema surrounding the appendiceal orifice (Figure 2).

**Figure 3:**
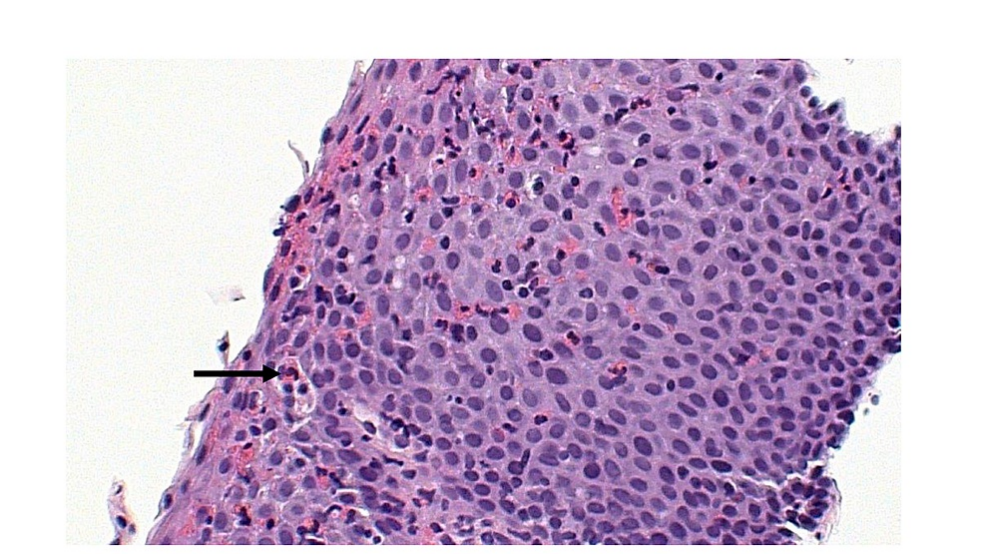
Histopathological appearance of esophageal epithelium with eosinophilic esophagitis. Esophageal epithelium with numerous eosinophils infiltration (black arrow).

**Figure 4:**
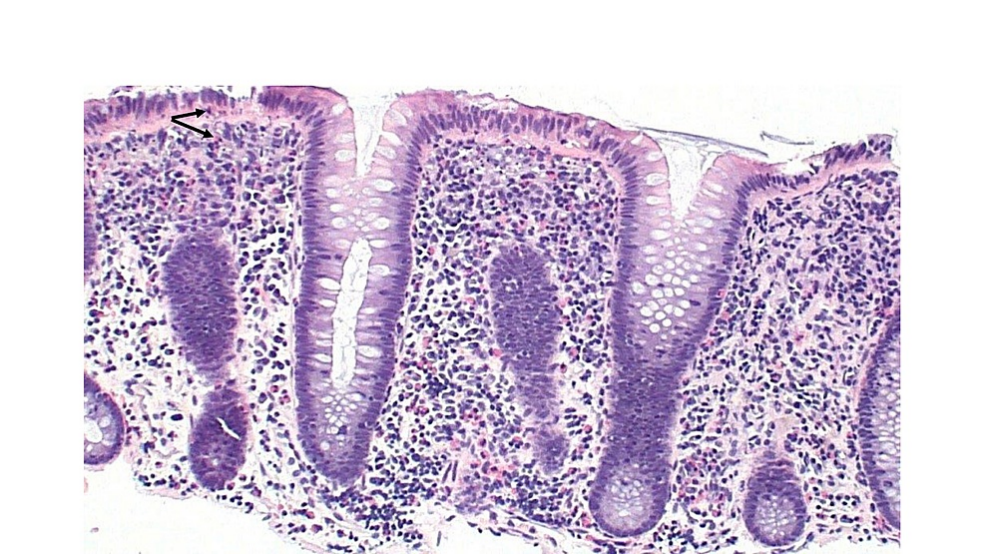
Histopathological appearance of ascending colon in a patient with eosinophilic colitis. Colonic mucosa with prominent eosinophils (>100 per high-power field) in the lamina propria and scattered intraepithelial eosinophils (black arrows).

On histopathology, samples from the esophagus revealed >80 eosinophils/hpf consistent with EoE (Figure 3). Biopsies from the ascending colon demonstrated eosinophils in the lamina propria and epithelium with >100 eosinophils/hpf (Figure 4). The EC cutoff at >100 eosinophils/hpf has been suggested [7]. The stomach and duodenum were macroscopically and histopathologically normal. He was diagnosed with EC. Shortly afterward, he received a 10-day course of oral steroids for severe acute respiratory syndrome coronavirus 2 (SARS-CoV-2) pneumonia, which ultimately relieved his diarrhea and cramping further supporting the diagnosis.

## Discussion

This case highlights an important aspect of EGID; patients with EoE and new GI symptoms require a high index of suspicion for the involvement of distal segments of the GI tract. EGID requires both clinical and histological investigation for diagnosis, highlighting the importance of luminal examination with endoscopic biopsies.

Although EGID can occur at any age, the majority of patients are children and adults <50 years old, affecting males more than females, with a predilection for Whites compared to other races [1-3]. Patients often present with abdominal pain, dysphagia, and vomiting that wax and wane. Approximately 70% of patients with EGID report a history of other allergic disorders, such as asthma, atopic dermatitis, or other seasonal and food allergies [8]. The pathogenesis appears to be a response to ingested and/or inhaled allergens mediated by T-helper 2 cells in the GI tract leading to eosinophilia, chronic inflammation, and GI symptoms [9]. Eosinophils are normally present within the rest of the GI tract, making the diagnosis of EGID in distal segments of the GI tract more challenging histopathologically, with the lack of clear threshold values for diagnosis [10]. Management of EGID focuses on symptomatic, endoscopic, and histological improvement.

The initial management of EoE is with either a food elimination diet or PPI therapy depending on patient preferences. PPI therapy can result in clinical response in two-thirds of patients, with PPI-refectory patients being treated with an elimination diet or swallowed topical steroids [11]. Dietary elimination involves the systematic elimination of common food allergens to determine the underlying culprit with the goal of achieving symptom resolution, histologic remission, and endoscopic improvement in select patients [12,13]. Both dietary elimination and PPIs can be used in combination to achieve a histologic and symptomatic response. If symptoms do not improve, options include a more restrictive elimination diet with or without topical steroids [13]. Systemic steroids can be used in refractory cases of EoE; however, data comparing topical and systemic steroids are sparse. A randomized clinical trial of 80 patients with EoE comparing oral prednisone versus topical fluticasone showed that oral prednisone resulted in a greater degree of histologic improvement with similar rates of symptom resolution [14]. Both topical budesonide and inhaled fluticasone have shown similar efficacy [15]. Newer agents targeting interleukin (IL) signaling are evolving for the treatment of EoE. Dupilumab, a subcutaneous monoclonal antibody targeting IL-4 receptors resulting in the disruption of IL-4 and IL-13 signaling, was studied in a multicenter, randomized, double-blind, placebo-controlled phase 2 clinical trial over a 12-week period and was associated with the clinical, histological, and endoscopic improvement compared to placebo. This study was limited by the small sample size, exclusion of patients with strictures, and the short duration of treatment and follow-up [16].

Unlike EoE, initial non-pharmacological treatments in EC, including dietary elimination measures, have been of limited effect in the adult population with mixed data [10]. Oral steroids remain the mainstay of treatment in many cases of EC [5]. However, other forms of steroids, including rectal budesonide, have been reported and should be considered given decreased systemic side effects [17]. Patients with persistent/relapsing episodes who require longer treatment course or maintenance therapy benefit from topical agents given the side effects of systemic steroids [17]. Our patient’s clinical course suggests a diagnosis of EC which responded to more aggressive therapy albeit prescribed for SARS-CoV-2. Enteric delayed-release or rectally administered steroid preparations may be a potential treatment option for patients who require long-term therapy.

Limitations of our paper include non-maximal steroid dosing per current guidelines and non-adherence to a full six-food elimination diet (soy). The patient was also treated primarily with serial dilations rather than a step-up or step-down approach to identify a primary food trigger. Inadvertent dietary non-adherence is also always a possibility. In addition, testing for systemic parasitic infections was not completed. Finally, the short-term follow-up limits our conclusions regarding long-term outcomes.

## Conclusions

In patients with EoE who develop new GI symptoms or symptoms refractory to medical therapy, evaluation for eosinophilic inflammation of the distal GI tract should be considered. In patients with confirmed EC or EGE, standard topical steroid formulations may not be effective, alternative formulations of steroids or a short course of systemic steroids should be considered. Further research is needed to parse the best long-term treatment strategy, perhaps enteric formulations designed for inflammatory bowel disease can be borrowed to treat this rare disease.
